# Focus-ING on DNA Integrity: Implication of ING Proteins in Cell Cycle Regulation and DNA Repair Modulation

**DOI:** 10.3390/cancers12010058

**Published:** 2019-12-24

**Authors:** Jérôme Archambeau, Alice Blondel, Rémy Pedeux

**Affiliations:** Université de Rennes, INSERM U1242, Chemistry Oncogenesis Stress Signaling (COSS), CLCC Eugène Marquis, 35042 Rennes, France; jerome.archambeau@univ-rennes1.fr (J.A.); alice.blondel@univ-rennes1.fr (A.B.)

**Keywords:** ING2, ING3, ING4, ING5, acetylation, p21, NHEJ, HR

## Abstract

The ING family of tumor suppressor genes is composed of five members (ING1-5) involved in cell cycle regulation, DNA damage response, apoptosis and senescence. All ING proteins belong to various HAT or HDAC complexes and participate in chromatin remodeling that is essential for genomic stability and signaling pathways. The gatekeeper functions of the INGs are well described by their role in the negative regulation of the cell cycle, notably by modulating the stability of p53 or the p300 HAT activity. However, the caretaker functions are described only for ING1, ING2 and ING3. This is due to their involvement in DNA repair such as ING1 that participates not only in NERs after UV-induced damage, but also in DSB repair in which ING2 and ING3 are required for accumulation of ATM, 53BP1 and BRCA1 near the lesion and for the subsequent repair. This review summarizes evidence of the critical roles of ING proteins in cell cycle regulation and DNA repair to maintain genomic stability.

## 1. Introduction

The Inhibitor of Growth (ING) gene family was first described in 1996 by the identification of p33ING1 in a study using a strategy based on Genetic Suppressor Elements whose expression promotes tumorigenesis by inhibiting the expression of Tumor Suppressor Genes [1]. Thereafter, ING2, ING3, ING4 and ING5 were identified by sequence homology.

**Figure 1:**
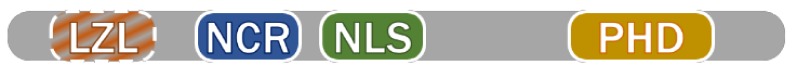
Linear representation of ING proteins sequence. The LZL motif (orange striped) that allows the interaction with other proteins is present on ING2, ING3, ING4 and ING5 but not ING1. The NCR domain (blue), specific to the ING family, is shared between all the INGs. The NLS domain (green) is responsible for the nuclear localization of the protein and the PHD motif (yellow) for the binding to methylated histones.

High homology has been observed between ING proteins and, although their N-terminus domain is unique for each family member, they share a very similar structure since four specific regions are found in the C-terminus of all five ING members: an LZL motif, an NCR, an NLS and a PHD motif (Figure 1). Indeed, ING proteins harbor an NLS domain favoring their targeting to the nucleus where INGs have been described to fulfill their main functions [2]. In addition, two interaction domains are present on the ING sequence, a LZL motif allowing interaction with proteins containing the Leucine Zipper motif [3] and a 50 amino acids PHD motif containing zinc binding domain already found in chromatin-interacting proteins and known to bind to methylated H3 histone tail [4,5,6]. In the case of ING proteins, the PHD motif has been mainly described to interact with H3K4me3 [7]. Finally, all ING members harbor a NCR domain which is very specific to this family and whose role is poorly described (the genomic structure of ING was reviewed in [8,9]). In addition, ING4 and ING5 crystal structures have shown that they can dimerize with each other thanks to the helical structure at their N-terminus that allows coil-coil folding and anti-parallel dimerization [10,11]. Homodimerization is necessary for their function [10]. Nevertheless, heterodimers of ING4 and ING5 have also been described, an interaction mainly due to the high sequence fidelity at their N-terminus (75%) [11]. The N-Terminus of ING5 is essential not only for its stabilization in dimers but also for its cellular localization because the N-Term mutants of ING5 are rather located in the cytoplasm than in the nucleus. Besides, partly because of its coil structure, the NLS domain of ING5 has been described as interacting with DNA [11].

The tumor suppressor functions of the ING genes have been established by clinical studies. ING1 has been reported to be negatively regulated at mRNA levels in breast, ovarian, lung, stomach, brain and lymphoid cancers. The ING1 protein is downregulated in melanoma, breast cancer, leukemia and in oral squamous cell carcinoma. Concerning ING2, mRNA levels were found to be downregulated in breast ovarian, lung cancer and hepatocellular carcinoma, whereas ING2 protein was described to be downregulated in melanoma. For all cancers in which they are negatively regulated, ING1 and ING2 protein levels decreased in the nucleus, suggesting a mutation closer to their NLS domain and/or an impaired interaction with a protein required to target ING1 and ING2 to the nucleus. It has been reported that ING3 was negatively regulated at mRNA level in squamous cell carcinoma of the neck and in hepatocellular carcinoma. ING3 protein, as well as its homolog ING2, has been shown to be downregulated in melanoma. ING4 mRNAs were downregulated in hepatocellular carcinoma, HNSCC and melanoma. In the latter two cases, ING4 has also been described as negatively regulated at protein levels in astrocytoma, breast cancer and lung cancer. Finally, mRNAs of ING5 are downregulated in oral cell squamous carcinoma (more details are reviewed in [12]). In most cases, decreased levels of ING proteins and mRNAs were associated with bad prognosis, in accordance with their tumor suppressor role. Several studies have revealed an endogenous regulation of ING expression by some miRNA such as miR153-3p for ING2 [13]. (Regulation of ING expression by miRNA and other ncRNA is better reviewed in [14]).

The role of ING proteins as a tumor suppressor has been originally described as gatekeeper. Gatekeepers participate in inhibition of proliferation and direct control of cell growth, in part through transcriptional activity [15]. However, recently caretaker functions have been reported for ING proteins. Caretakers are known to maintain genomic stability by directly modulating chromatin events [15].

Chromatin represents the core of cell information necessary for all the underlying processes. Among the cancer hallmarks described by Hannahan and Weinberg, genomic instability, replication stress and DNA damage play an important role in promoting tumorigenesis [16,17]. The integrity of the genome can be altered at different stages of cell life, such as cell cycle, DNA replication and damage, because at each step, errors could lead to mutations facilitating the occurrence of tumorigenesis. To counteract these threats, cells have developed checkpoints throughout the cell cycle as well as a conserved pathway that take charge of lesions and their repair, called the DNA damage response (DDR).

In this review, we will provide evidence of the critical role that ING proteins play in the nucleus. We will summarize all parts and stages of the cell cycle and DNA repair in which ING proteins are involved to ensure DNA integrity and genomic stability. Our work shows that by participating in both the cell cycle and DDR, the INGs are necessary to optimize the relationship and orchestration between these two pathways.

## 2. Chromatin Remodeling

The nucleosome, which is the core unit of chromatin, is an octameric structure composed of pairs of histone proteins H2A, H2B, H3 and H4 wrapped with DNA. This organized association limits access of regulatory proteins to chromatin. Hence, chromatin remodeling is a critical process for DNA accessibility of factors involved in DNA repair and replication. During the cell cycle, chromatin remodeling mostly occurs in G1/S and S phase where it facilitates the progression of the replication fork mediated by other factors [4]. Indeed, condensed chromatin is not accessible to proteins whereas relaxed chromatin is an open conformation that is easier to bind for replication or repair factors. The chromatin condensation is partly regulated by post-translational modifications of the histones present along the DNA notably through histones acetylation and the methylation. Histone acetylation by Histone AcetylTransferase (HAT) or demethylation by Histone Demethylase (KDM) are considered to be readouts for the “open” chromatin required for gene expression, cell cycle progression and DNA repair. Conversely, histone deacetylation by Histone Deacetylases (HDAC) and methylation by Histone MethylTransferases (HMT) are defined as hallmarks of “closed” chromatin facilitating gene silencing.

### 2.1. INGs Belong to Histone-Modifying Complexes

Deregulation of chromatin remodeling is considered as a feature of cancer [18,19]. In fact, downregulation of HAT or HDAC results in genomic instability due to abnormalities of the cell cycle and acute sensitivity to DNA damage [20,21,22,23]. ING proteins are part of several HAT or HDAC complexes and have been implicated in chromatin remodeling. Indeed, ING1 and ING2 have been identified as subunits of the mSin3A/HDAC complexes. ING3 is part of the hNuA4 HAT complex. ING4 and ING5 were both described as members of the HBO1 HAT complex, but only ING5 was found in the MOZ/MORF HAT complex [19].

### 2.2. ING1 Participates in H3 and H4 Acetylation

**Figure 2:**
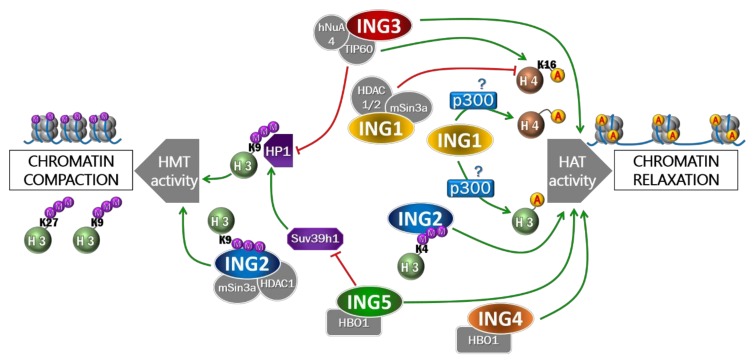
ING proteins participate in chromatin remodeling. Chromatin compaction is regulated by posttranslational modifications such as histone methylation (mostly recognized as a compacted chromatin hallmark), and histone acetylation (favoring chromatin relaxation). ING1, in complex with HDAC1/2, promotes acetylation of H3 and H4 histones. This acetylation is also due to ING1-induced p300 activity. ING2, which can bind either H3K4me3 or H3K9me3, promotes respectively HAT activity or HMT activity. Conversely, ING3, as a member of hNuA4 complex, promotes HAT activity, notably thanks to TIP60 whose HAT activity is ING3-dependent, but also participates in inhibiting HMT activity via HP1 chaperone protein. ING4 and ING5 (both in complex with HBO1) were described as HAT promoting factors. ING5 was also described to inhibit the HMT Suv39h1 and the subsequent histone methylation.

The two most expressed isoforms of ING1 in tissues, p47ING1a and p33ING1b, play a role in H3 and H4 acetylation. However, they exert different roles since ING1a overexpression leads to a decreased histone acetylation whereas ING1b overexpression induces H3 and H4 hyperacetylation [24]. In addition, ING1a is rather involved in senescent cells pathways [25,26]. For these reasons, we will focus here on ING1b. Increased binding of ING1b to chromatin may facilitate histone H4 acetylation by its associated HAT activity [27]. However, ING1b participates in the acetylation of both H3 and H4 [24,27]. In the case of UV-induced DNA damage, ING1b is essential for the acetylation of H3 and H4 because of its interaction with p300; furthermore, it is suggested that the latter cooperates with p53 for the acetylation of H3 and the relaxation of chromatin in response to UV [27,28] (Figure 2). Additionally, the recruitment onto chromatin of SWI/SNF, a chromatin remodeling complex, and the resulting “relaxed” conformation of chromatin were dependent on p300 histone acetylation [20]. Since ING1 [27] and ING2 [29] interact with p300, we can hypothesize that they participate in chromatin acetylation by p300.

### 2.3. The Dual Role of ING2 in Chromatin Remodeling

Although ING1 and ING2 belong to the mSin3A/HDAC complex, they can’t be present simultaneously in the complex since they are mutually exclusive [28]. Interestingly, when ING2 is part of the complex, it produces a different effect than ING1. ING2 has been shown to be part of the corepressor complexes mSin3A/HDAC1/2 and facilitates the activity of HMT. By binding H3K4me3 via its PHD domain, ING2 can bridge the mSin3A/HDAC complexes with chromatin [7,28,30]. The recruitment of mSin3A/HDAC1 leads to the repression of genes by hypermethylation or by increasing the activity of HDAC, especially on the promoter of cyclin D1, which results in its repression [7,30] (Figure 2). ING2 has been reported to be critical for HDAC activity of the mSin3A/HDAC complex because HDAC inhibitors induce ING2 dissociation from the complex, resulting in decreased HDAC activity [31]. Interestingly, the impact of ING2 on HMT activity was found to be dependent on the methylation status of H3 on Lys4 and Lys9. The methylation of H3K4 reduces ING2-related HMT activity whereas methylation on H3K9 enhanced ING2-associated HMT activity [30]. In addition, ING2 has been shown to be associated with several SWI/SNF complex proteins, suggesting that its involvement in chromatin remodeling may be related to SWI/SNF functions [19].

### 2.4. ING3 in Complex with hNuA4 Promotes Histones Acetylation

The acetyltransferase activity of the hNuA4 complex (in which ING3 is known to be part) has been shown to be essential in several cellular processes since it is involved in H4 acetylation. This modification is necessary for accurate replication and repair of DNA upon fork breakage or DNA damage [32]. The hNuA4 complex has also been described as participating in one of the first steps of chromatin remodeling in response to Double Strand Break (DSB). The hNuA4 subunit p400, which has ATPase activity, will promote the H2A.Z exchange on chromatin, a step required for TIP60 activity to acetylate H2A.Z as well as histone H4 to create relaxed chromatin domains and facilitate the recruitment of proteins involved in DNA repair [33]. Downregulation of the TIP60 HAT activity on H4 suppresses repair of DSB in mammalian cells, whereas depletion of TRRAP (HAT cofactor) decreases UV-induced hyperacetylation of H4 and recruitment of repair proteins at DSB sites [27,34]. In addition, impaired H4 acetylation is observed in *yng2*-deficient yeast cells (yeast homolog for ING3), demonstrating that the integrity of the NuA4 complex is essential for its HAT activity [35] (Figure 2). 

Other studies in *Saccharomyces cerevisiae* revealed that that Esa1-mediated histone H4 acetylation (yeast ortholog for TIP60) not only requires its recruitment (enhanced by Arp4, yeast ortholog for BAF53a), but also requires several NuA4 critical units such as Yng2, Tra1 and Arp4 (respectively ING3, TRRAP and BAF53a in mammals) and results in a decompaction of chromatin [36,37]. Since it has been shown that BAF53a is in the same complex as ING3 (like Arp4 with Yng2 in yeast cells) and because of its membership in the SWI/SNF complex, we can assume that these two complexes act in concert to remodel the chromatin.

Furthermore, the SWI/SNF complex is involved in chromatin remodeling after DNA damage. The SWI/SNF complex which participates in H2AX phosphorylation and interaction with acetylated nucleosomes, was reported to be recruited in a hNuA4-dependent manner [38]. Indeed, in concert with the HAT Gcn5, the hNuA4 complex is required for the acetylation of nucleosomes notably thanks to ING3 [38,39]. 

### 2.5. ING4 and ING5 Enhance Histones Acetylation

Through its membership in the HBO1 HAT complex, ING4 has been described as participating in the acetylation of H4 as well as H3 to a lesser extent (Figure 2). Indeed, the acetylation of H4 by HBO1-ING4 is carried out on Lys5, Lys8, Lys12 but not Lys16 which is preferentially acetylated by the ING3-TIP60 complex. It should be noted that ING4-dependent histone acetylation affects cell cycle progression [19].

It has been reported that ING5 participates in chromatin remodeling in a p300-dependent manner. The acetylation of histone marks H3K18 and H4K16 have been shown to be dependent on ING5 since overexpression of ING5 enhances both H3K18ac and H4K16ac whereas the downregulation of ING5 inhibits this effect [40]. Acetylation of H4K16 is a crucial component of chromatin since its acetylated K16 residue is one of the first element to anchor H4 on the adjacent nucleosome, but also because acetylation reduces electrostatic interactions between H4 tails in the nucleosomes [41]. On the other hand, it has been described that H3K18ac was linked to gene expression [42]. ING5 in complex with HBO1 has been shown to antagonize Suv39h1, a HMT that enhances chromatin-compacted status by methylation of H3K9me3 [43] (Figure 2). Since HBO1 and ING5 were reported to participate in the early stages of replication by interacting with ORC1 and MCM helicases, two proteins involved in the pre-replication complex [19], we can hypothesize that the ING5/HBO1 complex keeps the chromatin open. This is supported by the inhibition of Suv39h1 by ING5/HBO1, that avoids the trimethylation of H3K9, described as being present at late replication sites and to enhance the assembly of heterochromatin [44]. However, it has been reported that ING5 binds to H3K4me3 through its PHD, thus enhancing the stability of the MOZ/MORF complex at promoters of activated genes [45].

## 3. Cell Cycle Regulation

Cell cycle regulation and DNA replication are well-controlled processes that prevent genomic instability. During its replication, the cell harbors various control checkpoints that are gates to prevent improper DNA synthesis or rearrangement that could lead to mutations. Uncontrolled cell proliferation is defined as a hallmark of cancer and may be the consequence of impaired checkpoints due to unbridled replication factors [46]. Several ING proteins have been described as participating in cell cycle regulation, notably through their interaction with p53, a guardian of the genome, one of the most described regulatory proteins.

Since the ING gene family has been identified and characterized to slow cell growth [1], their regulatory impact on cell cycle and cell replication is not surprising. Subsequently, the involvement of ING in the regulation of the cell cycle has been extensively described. In this part, we will focus on the role of ING proteins on cell cycle regulation in the absence of damages.

### 3.1. ING1 in Cell Cycle Regulation

**Figure 3:**
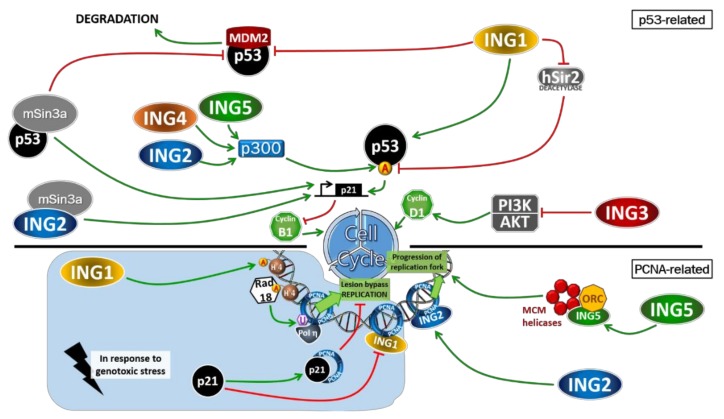
ING proteins regulate cell cycle in a p53-dependent and independent manner. Cell cycle is negatively regulated by INGs in various ways. UPPER PART: ING1 by interacting with p53 promotes its activation of p21. By inhibiting both deacetylase (hSir2) and degradation of p53, ING1 stabilizes p53. In addition, Sin3a can interact with p53 and stabilize it, then inhibiting MDM2-associated degradation of p53. ING2, ING4 and ING5 can induce negative cell cycle regulation through p300 activation leading to the acetylation of p53. In addition, ING2 in complex with mSin3A can directly activate p21 expression thus inhibiting cell cycle independently of p53. Finally, ING3 by inhibiting the PI3K/AKT activation, can decrease the expression of cyclin D1 resulting in cell cycle arrest. LOWER PART: in response to DNA damage or genotoxic stress, ING1 can facilitate lesion bypass and further replication by promoting Rad18 loading onto chromatin. In a more physiological way, ING2 interaction with PCNA promotes the progression of the replication fork onto chromatin. ING5 also helps this progression by interacting with the MCM helicases, then preventing replication fork collapsing or replicative stress.

ING1, which was the first ING gene identified, was also the first described to play a role in cell cycle regulation. p33ING1b negatively regulates the cell cycle because its overexpression inhibits cell cycle progression [47], highlighted by a reduced ability to form colonies [48]. Another piece of evidence of its involvement in cell cycle regulation is that the expression of ING1 is regulated during the cell cycle; its expression increases from the late G1 phase to the S phase where it reaches its maximum and then decreases in the G2 phase. This modulation suggests a major role in the cell cycle, notably in the G1/S phase [49]. Indeed, the cells positively regulated for ING1b harbor a p53-dependent cycle arrest in G1-phase, an effect also described in ING2-upregulated cells [50]. ING1b downregulation induced a more rapid progression from G1 to G2/M [51] confirming that ING1b participates in cell cycle regulation by modulating p53 anti-proliferative activity due to their physical interaction [50,52,53,54]. ING1b-dependent cell cycle arrest was reported to be enhanced through increased p53 transcriptional activity since the p21 promoter was targeted and subsequently induced a cell cycle arrest [50] (Figure 3). Since ING1b can bind p53 to the same region as MDM2, it inhibits MDM2-p53 interaction, abrogates p53-degradation and increases its stability [52]. This increased stability of p53 is also promoted by ING1b ability to bind ubiquitinated p53 thanks to a Ubiquitin-Binding Domain in the ING1b sequence [55]. p19/ARF which also participates in MDM2-p53 degradation was reported to interact with ING1b thus facilitating its targeting to the nucleolus for cell cycle regulation. 

In addition, cell cycle arrest induced by overexpression of ING1b is impaired in ARF-deficient cells [52,56]. Finally, since it has also been shown that mSin3A binds to p53, which will further enhance its stabilization [57], and since ING1b has been described as part of the mSin3A complex, we can hypothesize that ING1b assists the loading of mSin3A on the chromatin and acts in concert with it to enhance the cell cycle arrest by regulating the transcriptional activity of p53.

However, ING1b has been described to regulate the cell cycle in a p53-dependent or independent manner. It has been shown that ING1b delays cell growth even in the absence of p53, thanks to its PHD domain which recognizes H3K4me3 [54,58]. ING1b has also been described to regulate the cell cycle in response to UV and replicative stress, which will be discussed in the next part.

### 3.2. ING2 in Cell Cycle Regulation

Similar to its homolog ING1, the expression of ING2 is also regulated throughout the cell cycle. Indeed, its expression constantly increases from the early S phase to the G2/M phase which is correlated with the increased expression level of p21 [59]. ING2 was also reported to inhibit the cell cycle and the ability to form colonies [50]. ING2 exerts this effect particularly at the G1/S transition where it regulates the expression of p21 (a p53 transcriptional target). In cells silenced for ING2, an accelerated progression in the G1 phase was observed, associated with a decrease in the expression of p21 [59]. Unlike its counterpart ING1, ING2 is not able to bind p53 [50]. Thus, ING2 can act on cell cycle in a p53-dependent manner by the acetylation of p53 notably thanks to p300 acetyltransferase activity [29,50,60] (Figure 3). In contrast to ING1b, whose overexpression does not enhance p53 acetylation, overexpression of ING2 leads to an increase in p53 acetylation on Lys-382 resulting in increased stability of p53. Silencing of ING2 leads to a proportional decrease in the level of p53 acetylation [50]. In addition, it is known that this acetylation site targeted by p300 HAT is involved in p53 activity [61]. However, as observed with ING1, ING2 can regulate the cell cycle in a p53-dependent or independent manner, as the expression of p21 was regulated by ING2 even in cells lacking p53 [59]. Furthermore, ING2 has also been described as a participant in the negative regulation of the cell cycle by inhibiting the expression of the cyclin D1 gene independently of p53 [7]. This effect is most likely due to its mSin3A-associated HDAC activity that promotes repression of gene expression. In both cases, recognition of H3K4me3 by the ING2 PHD domain is required for activation of transcription [7,62]. It has also been reported that the PHD domain of ING2 was essential for the regulation of the p53 pathway since phosphatidylinositol 5-phosphate (PtdIns (5) P) could interact with ING2 as a nuclear receptor by binding to its PHD and PBR and favoring ING2-dependent cell cycle inhibition by p53 acetylation and increased expression of p21 [63,64].

Even if no PIP domain is present on the ING2 sequence, it can interact with PCNA through a region located between the LZL and NLS of ING2 [65]. This interaction is necessary for PCNA-mediated cell cycle regulation by ING2. In fact, ING2 targets PCNA at the chromatin fraction during replication to ensure the progression of the replication fork. As a result, cells deficient for ING2 exhibit a reduced amount of PCNA bound to the chromatin fraction associated with a slower replication fork progression [65] (Figure 3).

Finally, ING2 can participate in the regulation of the cell cycle at different stages and through different interactions, but its effects could also depend on other factors such as PtdIns (5) P [63]. In addition, it has also been reported that ING2 participates in the inhibition of TGF-mediated cell proliferation, since the ability of TGF-β to promote cell cycle arrest is enhanced by the overexpression of ING2, probably due to its PHD domain [66].

### 3.3. ING3 in Cell Cycle Regulation

Evidence has shown that ING3 participates in the regulation of the cell cycle because its overexpression leads to proliferative defects [67,68,69,70]. Like other ING members, ING3 was found to negatively regulate cell growth in a p53-dependent manner since its overexpression not only inhibited cell proliferation but also colony formation, except in cells inactivated for p53 [19,36,67,71,72,73]. The overexpression of ING3 has been described as decreasing the number of cells in the S phase [67]. More recently, it has been reported that ING3 negatively regulates the cell cycle. This effect is more likely related to PCNA since PCNA mRNA levels decreased in the cells up-regulated by ING3 [74]. Indeed, PCNA has been described as interacting with Cdc25C rather than p21 to promote the G2/M transition [75,76]. Thus, the overexpression of ING3 inhibits PCNA expression and the subsequent G2/M transition. Taking all these results into account, ING3 seems to be involved in the G1-S phase transition. Dependence on p53 has been demonstrated because ING3 can increase the activity of the p21 promoter [19]. In addition, ING3 has been shown to inhibit the activation of PI3K and AKT, whose inhibition has already been described to promote cell cycle arrest. In addition, a downregulated transcription of Cyclin D1 was observed in ING3 upregulated cells [74,77,78] (Figure 3). Cyclin D1 was already described to be regulated by PI3K [79,80]. Taken together, we can hypothesize that the inhibition of the cell cycle by ING3 could be mediated by PI3K.

However, the involvement of ING3 in the cell cycle is more ambivalent because in some cases ING3 could induce rather than inhibit cell growth, as it has been observed in the *Yng2* mutant yeast that harbors a mitotic delay [35]. Indeed, an increase in the expression of ING3 has been shown to correlate with proliferation in rapidly proliferating and rapidly replenishing cells such as those in the small intestine, bone marrow and epidermis [81], but also to increase the levels of proliferation marker when it is overexpressed in prostate cancer cells [70]. In the same study, the exogenous expression of ING3 increases colony formation; a result which corroborates what was observed in our recent study where the tumor cells downregulated for ING3 exhibited a reduced capacity for colony formation under physiological conditions (without genotoxic treatment) in both yeast and human cells [82]. Since TIP60 has been shown to activate cell cycle without being part of the hNuA4 complex, the opposite effect of ING3 on cell cycle could depend on its interaction with the hNuA4 complex. Because ING3’s role differs in some cell types such as prostate tumor cells, we can also hypothesize that the results obtained in different cell types do not depend only on the mutation status of ING3 but also on the related and surrounding pathways that could be mutated on their own and then differentially regulate ING3 or its effects [83].

### 3.4. ING4 and ING5 in Cell Cycle Regulation

ING4 and ING5 are also negative cell cycle regulators because their overexpression reduces colony formation in a p53-dependent manner. Indeed, p53 transcriptional activity is favored by ING4 and ING5, in particular on the p21 promoter whose activity is enhanced when ING4 and ING5 are overexpressed [84]. The expression of p27 and cyclin D1 (two cell cycle regulators) were regulated by ING4 to promote cell cycle arrest, upregulate p27 and downregulate cyclin D1 expression when ING4 is overexpressed [85] (Figure 3). Furthermore, ING4 and ING5 have been described as promoting p53 transcriptional activity by increasing its acetylation in particular by physically interacting with the p300 acetyltransferase and by enhancing its activity with respect to p53. As observed for ING1b, physical interactions between p53 and ING4 or ING5 were also observed [84].

Through their p53-mediated cell cycle regulation, ING4 and ING5 facilitate the transition from G1 phase to S phase since a reduced cell population was observed in S phase when ING4 and ING5 are overexpressed while the proportion of cells in G1 increased [84]. More recently, the role of ING5 in the acetylation of p53 has been confirmed as ING5 promotes the autoacetylation of p300, resulting in its activation and the associated acetylation of the p300-targeted proteins. Indeed, p53 acetylation on Lys382 was enhanced by ING5 overexpression resulting in the subsequent expression of p21 [40]. ING5, as a subunit of a HAT complex, was also described to be a cofactor of TIP60 and to favor p53 acetylation to promote p21 expression [86]. In summary, ING4 and ING5 regulate the transition from G1 to S in a p53-dependent manner by interacting with p53 and by inducing p300-mediated acetylation of p53 (two roles shared with ING1b and ING2).

It has been reported that ING4 was regulated through its citrullination by the peptidylarginine deminase 4 (PAD4) [87]. Indeed, PAD4 has already been described as influencing gene expression by citrullination of H3 (conversion of peptidyl arginine to citrulline) [88]. PAD4 has been described as citrunillating ING4 on the same region that interacts with p53 therefore inhibiting ING4-p53 interaction responsible for p21 expression and cell cycle arrest. In addition, it has been shown that citrullination of ING4 facilitates degradation of ING4 by the proteasome [87]. Thus, ING4-dependent cell cycle regulation could be mediated by PAD4.

More recently, it has been reported that CBP/p300 acetylates PCNA on four lysines (Lys13,14,77 and 80) in response to UV-induced DNA damage to promote NERs. p300-mediated acetylation induces PCNA degradation and then promotes genomic stability by recruiting specific NER factors [89]. Since ING1, ING2, ING4 and ING5 can regulate p300 activity during the cell cycle, we can hypothesize that in response to DNA damage, they could eliminate chromatin-bound PCNA by promoting its p300-mediated acetylation.

## 4. DNA Repair Regulation

Every day, cells are exposed to hundreds of DNA damages which dangerously threaten the integrity of DNA. In addition, the integrity of chromatin is threatened by endogenous or exogenous factors resulting in DSBs and various other damage such as oxidative damage, depurination, etc. To prevent mutations and tumorigenesis associated with unrepaired DNA damage, cells have a coordinated pathway called the DNA Damage Response (DDR). The DDR is conserved from yeast to human. This response consists of the regulation of proteins to sense the lesion, signal its presence and initiate appropriate repair of DNA damages through recruitment of repair complexes. In addition to proper repair of DNA, the DDR also includes the cell cycle regulation after DNA lesions, as described below for ING1b. Depending on the phases of the cell cycle and the type of damage, repair will be performed either by nucleotide excision repair (NER), base excision repair (BER), mismatch repair (MMR), Inter-strand Cross Linking Repair (ICL) or Trans-Lesions Synthesis (TLS) in response to bases lesions. In more severe cases such as breaks, repair is driven by Single Strand Break Repair (SSBR) or Double-Strand Break Repair (DSBR) that encompasses Non-Homologous End Joining (NHEJ) or Homologous Recombination (HR) that manage the repair of DSBs [90]. Because actors for the DDR are among the most frequently mutated genes in tumors [16], involvement of ING proteins in response to DNA damages is not surprising.

### 4.1. ING1 and ING2 Participate in UV-Induced Repair

**Figure 4:**
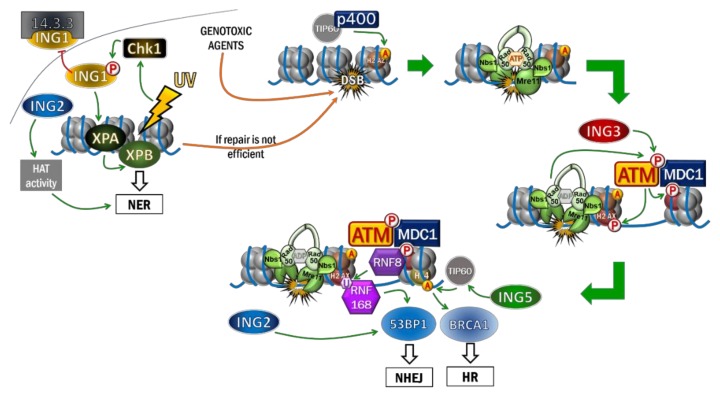
ING proteins are involved in the DNA Damage Response. ING1 is involved in UV damage: activated after UV damage, Chk1 phosphorylates ING1 on Ser126 which will stabilize it. ING1 is then able to promote XPA recruitment and subsequent DNA repair by NER. ING2, which facilitates NER, is also required in DSB repair in which it participates in 53BP1 accumulation at DSB sites. ING3 participates in the DDR pathway by promoting ATM activation and the signaling pathway that finally leads to the accumulation of 53BP1 or BRCA1 then initiating repair by either NHEJ or HR respectively. ING5 was described as a TIP60 cofactor that promotes BRCA1 accumulation thanks to H4K16 acetylation.

The first indication of ING’s involvement in response to DNA damage has been reported with ING1b since cell cycle and apoptosis can be regulated in a ING1b-dependent manner in response to UV lesions [51,91,92]. The repair mechanism involved after UV-induced DNA damage is preferentially NER. This pathway leads to DNA repair following recruitment and activity of several factors such as XPA, XPB, RPA and also PCNA [93,94,95] (Figure 4). 

In response to UV-induced DNA damage, PCNA activity and recruitment onto chromatin were in part regulated by ING1b [51,91]. Moreover, the PCNA-binding protein (PIP) domain present on the N-Term region of ING1b has been described to allow interaction with PCNA and then modulate the cell cycle in response to UV [51,91]. The CDK inhibitor p21 also has a PIP domain that has been described to interact with PCNA. Therefore, p21 through its PIP domain may compete with ING1b and inhibit its interaction with PCNA, resulting in cell cycle arrest [91].

On the other hand, ING1b was described to maintain genomic stability by promoting PCNA loading on chromatin in response to replicative stress. Indeed, ING1b was shown to promote Polη-mediated lesions bypass. Upon replicative stress the replication fork progression mediated by PCNA is impaired in S phase. Then, ING1b promotes the acetylation of H4 which leads to the recruitment of the ubiquitin-ligase Rad18 on the chromatin, which results in a monoubiquitination of PCNA. Polη, a DNA polymerase that is able to replicate across UV lesions, preferentially binds monoubiquitinated PCNA and overcome replication fork stalling [51] (Figure 3).

In addition, ING1 knock out mice are more sensitive to γ irradiation [96]. Subsequently, studies have shown that after UV exposure the expression of ING1b was increased but that its cellular location was also modulated since ING1b was translocated into the nucleus by the Nucleolar Targeting Sequence (NTS), to induce apoptosis [91,92,97]. Besides, the effect of ING1b in DNA repair seems to be dependent on Chk1, a kinase activated in the DDR by ATM/ATR kinases. In addition, Chk1 has been reported to phosphorylate ING1b on Ser126 in response to Doxorubicin and UV, enhancing its nuclear localization by disrupting the interaction between ING1b and 14-3-3 [98,99]. However, ING1b remains dispersed in the nucleus instead of being recruited at the level of UV-induced DNA damage [27]. It has been reported that the role of ING1b in response to UV-induced DNA damage enhances the NER pathway independently of the functional status of p53. Indeed, ING1b can promote the recruitment of XPA on chromatin after UV irradiation, as well as the recruitment of XPB, two factors involved in NER and known to stabilize open DNA complexes [27]. Finally, it was reported that ING1b enhanced NER by interacting with GADD45, through its PHD motif, and with SAP30, through a SAP30-interacting motif [27,100].

ING1b also contributes to repair upon UV-induced DNA damage by facilitating chromatin relaxation notably because it interacts with p300 to enhance H3 and H4 acetylation [27]. Histone hyperacetylation (induced by the HDAC inhibitor, TSA) can overcome the effect of the depletion of ING1b on the NER, demonstrating that the effect of ING1b on the repair of UV-induced damage is due to its role on the activity of the HAT [27].

Although the first involvement of ING1b in DNA repair has been described in response to UV-induced damage, other studies have shown a role for ING1b in the response to other DNA damaging agents. In fact, ING1b reduces cellular sensitivity to DNA damage caused by doxorubicin [54]. More recently, it has been clearly demonstrated that ING1b is involved in DNA repair because its overexpression improves repair after UV damage, while its downregulation impaired DNA repair in response to various DNA damaging agents (UV, neocarzinostatin (NCS), IR and H_2_O_2_). In addition, γ-H2AX signal, a read-out of DNA breaks, remains at high levels a long period of time after DNA damage induction in cells downregulated for ING1b [97]. 

Later, it was reported that ING2 was involved in UV-induced DNA repair in the same way as ING1b [101]. Indeed, even if ING2 is not recruited to the sites of lesions after UV irradiation of melanoma cells, it enhances the repair of UV-damaged DNA in a p53-dependent manner. ING2 has been reported to induce histone H4 hyperacetylation in response to UV exposure [101]. This ING2-dependent HAT activity leads to the relaxation of chromatin and the recruitment of XPA to the sites of UV lesions to ensure NER [101] (Figure 2; Figure 4). As described by Adamson and colleagues, the role of ING1b in the DDR is focused primarily on the NERs in response to UV damage, while ING2 and ING3 would be more involved in the DSB repair [102].

Finally, in response to etoposide-induced DNA damage, ING2 has been described as regulating more than 200 genes involved in cell cycle progression and cell proliferation. This effect has been shown to be associated with PtdIns (5) P. This interaction stabilizes the binding of ING2 to the promoter of genes via its binding to H3K4me3 [103]. One hypothesis is that ING2, in response to DNA damage can regulate cell cycle until the repair is achieved.

### 4.2. DSB Repair (DSBR) Is Modulated by ING Proteins

DSBs are the most serious form of DNA damage because their repair is essential to the integrity of DNA and can lead to mutations if the repair is inappropriate. The DDR pathway for repairing DSB results in the recruitment of 53BP1 and BRCA1, two effectors that will direct repair NHEJ or HR.

Mre11-Rad50-Nbs1 complex (MRN) is one of the first actor of the DDR that senses free ends of chromatin lesion sites. Each member is associated in dimers forming a heterohexameric complex that sits on DNA [104,105,106]. Mre11 possesses an endonuclease activity whereas Rad50 is an ATPase. ATP hydrolysis by Rad50 induces an ‘open’ conformation of the complex that reveals the DNA nuclease site of Mre11. Conversely, in ATP-bound conformation, Rad50 blocks the Mre11 nuclease active site and promotes ATM activation [107]. ATM is a serine/threonine kinase associated in inactive dimers whose N-Terminal domain physically interacts with the C-terminus of Nbs1 [108]. As a result of the interaction with MRN, ATM will autophosphorylate resulting in active monomers [109,110]. ATM activation may also require HAT activity of TIP60 that binds H3K9me3 surrounding DSB through its chromodomain [111]. Once activated, ATM phosphorylates histone variant H2AX on Ser 139 in the vicinity of the damaged site and then organizes platforms of phosphorylated H2AX (γH2AX) that will favor the recruitment of downstream DDR factors [112,113] (Figure 4). ATM recruitment favors recruitment of MDC1 that can bind ATM via its FHA N-terminal domain, and γH2AX through its C-Terminal BRCT domain, thus creating a positive feedback loop. Indeed, thanks to this interaction, ATM-dependent phosphorylation of H2AX is amplified since activated ATM is accumulated at DNA damage sites [114]. Then, the E3 ubiquitin ligase RNF8 is recruited in the vicinity of DSB by binding phosphorylated MDC1 [115]. RNF8 ubiquitylates H2A and H2AX. By initiating the ubiquitylation of histones, RNF8 increases the local concentration of RNF168 and physically recruits this ubiquitin ligase. RNF168, by participating and increasing ubiquitylation of H2A and H2AX, promotes the recruitment of 53BP1 and BRCA1 [116,117].

### 4.3. ING2 Can Regulate DSBR

The involvement of ING2 in response to DNA damage was first published by Nagashima and collaborators, showing that ING2 expression was induced after genotoxic treatment by etoposide and NCS [50]. This study also shows that the induction of ING2 is dependent on the cell damaging agent since X-rays, doxorubicin bleomycin or cis-platinum didn’t enhance ING2 expression. ING2 has also been shown to promote p53-acetylation and the subsequent increase in p21 expression after etoposide-induced DSB suggesting a role for ING2 in cell cycle regulation in response to DNA damage [63]. In addition, ING2 has been implicated in maintaining genomic stability since an increase in H2AX phosphorylation (γH2AX) was observed in siING2 cells without genotoxic treatment [65]. Increased phosphorylation of Chk1 has also been reported, a factor phosphorylated by ATM/ATR kinases in the DDR pathway activating an intra-S phase checkpoint [65]. ING2 was found to promote 53BP1 recruitment at DSB sites [118] (Figure 4). Indeed, ING2 is required for chromatin location of the E3 ubiquitin ligase RNF168 [119]. RNF168 is a known DDR factor that ubiquitylates H2A histone and 53BP1 to promote its recruitment and response to DSB [120,121,122].

### 4.4. ING3 Modulates DSBR

The first evidence of ING3’s caretaker role was discovered in yeasts as cells lacking Yng2 protein (Yng2p) lost their NuA4 activity and were more sensitive to temperature [35]. In the same study the NuA4 complex was present without Yng2p, but had no HAT activity suggesting that Yng2p stabilizes or activates NuA4 [35]. In addition, depletion of ING3 in mammalian cells leads to an impaired repair of DNA damage after irradiation since γH2AX foci resolution is delayed compared to control cells [123]. Consistent with these reports, we have shown recently that yeast cells deficient for *Yng2* exhibit increased sensitivity to various DNA damaging agents [82]. This effect was due to involvement in the DDR since ING3 is recruited in the vicinity of DSB. ING3 was shown to be essential for ATM activation which is, as discussed above, a major actor of the DDR. The defective phosphorylation of ATM observed in ING3-silenced cells could be due to an impaired interaction between the MRN complex and ATM (Figure 4). As a result, the recruitment of many factors of the pathway is ING3-dependent since both RNF8 and RNF168 accumulations are impaired in ING3-silenced cells. To the same extent, ING3 participates in the subsequent recruitment of 53BP1 and BRCA1, which are required for the effective repair of DNA. The impaired recruitment observed for both BRCA1 and 53BP1 in ING3 silenced cells was associated with deficient repair either by HR or NHEJ [82,102]. 

Since ING3 is involved in chromatin remodeling and RNF168 accumulation depends on modified histones, we can suppose that defective RNF168 accumulation and subsequent 53BP1 and BRCA1 activation is not only due to impaired ATM signaling but is also a consequence of altered chromatin remodeling after DNA damage. Interestingly, DNA-PKcs has recently been described to regulate ATM signaling. Indeed, when the kinase activity of DNA-PKcs is activated by phosphorylation on Ser2056, it can phosphorylate ATM on multiple sites and inhibit its activity [124]. We can then hypothesize that ING3 plays an inhibitory role on DNA-PKcs activity and indirectly favors the activation of ATM. More recently, the involvement of UFMylation, a new lysine post- translational modification, was described in the DNA damage response. UFL1, an E3 ligase allowing UFMylation, has been described to participate in the early steps of the DDR by UFMylating Mre11 and histone H4, then promoting the activation of ATM and a further positive feedback loop [125,126]. In addition, it has been reported that monufmylation was essential for the subsequent recruitment of TIP60 and activation of ATM [126]. So, we can hypothesize that ING3 could participate in ATM signaling through modulating UFL1 recruitment at DSB sites and promoting UFMylation of Mre11 and histone H4. 

### 4.5. ING4 and ING5 Involvement in DDR

Although the involvement of ING4 and ING5 in the response to DNA damage is less reported ING5’s role in DNA damage was highlighted by a study that showed an increased expression of ING5 in response to DNA damages [86]. Furthermore, DNA damage can impact on the subcellular localization of ING5 that translocates into nucleus upon doxorubicin treatment. As discussed above, ING5 was also shown to act as a cofactor with TIP60 to participate in p53 acetylation in response to DNA damage [86]. Thus, ING5 could also participate in the early steps of the DSBR by facilitating both H4 acetylation and ATM signaling in a TIP60-mediated manner. Besides, ING5 is shown to be necessary for p53-mediated apoptosis in response to DNA damage induced by doxorubicin. However, while early steps and epigenetics modifications can be modulated, another study reported that neither ING4 nor ING5 were involved in DSB repair since no alteration of HR repair was observed after ING4 and ING5 downregulation [102].

## 5. Conclusions

To conclude, we have reviewed here evidence of the crucial role played by the INGs in the nuclear processes, notably thanks to their optimized structure for various interactions with the histone marks, p53, p300, PCNA and also 53BP1. Interactions between cell cycle and DNA damage response occur between proteins common to both pathways in order to enhance efficiency of one pathway and prevent alteration of the other. For example, the cell cycle inhibitor p21 has been shown to interact with PCNA during DNA repair to prevent the interaction between p300 and PCNA to inhibit p300 HAT activity [127]. Since ING proteins have been described to be involved in both cell cycle regulation and DNA damage response, we can hypothesize that they play a pivotal role between these two pathways that is crucial for maintaining chromatin integrity and genome stability. ING proteins through their caretaker role and gatekeeper functions could be critical actors in the tumorigenesis process. By regulating pathways involved in genomic stability, INGs are promising targets in cancer treatment to optimize tumor cells sensitivity toward therapies.
